# Case-control study of mammographic density and breast cancer risk using processed digital mammograms

**DOI:** 10.1186/s13058-016-0715-3

**Published:** 2016-05-21

**Authors:** Laurel A. Habel, Jafi A. Lipson, Ninah Achacoso, Joseph H. Rothstein, Martin J. Yaffe, Rhea Y. Liang, Luana Acton, Valerie McGuire, Alice S. Whittemore, Daniel L. Rubin, Weiva Sieh

**Affiliations:** Division of Research, Kaiser Permanente Northern California, Oakland, CA 94612 USA; Department of Health Research and Policy, Division of Epidemiology, Stanford University School of Medicine, Stanford, CA USA; Department of Radiology, Stanford University School of Medicine, Stanford, CA USA; Departments of Medical Biophysics and Medical Imaging, Sunnybrook Research Institute, University of Toronto, Toronto, Canada; Smarter Imaging Program, Ontario Institute for Cancer Research, Toronto, Canada

**Keywords:** Breast cancer, Mammography, Mammographic density, Risk factors, Epidemiology

## Abstract

**Background:**

Full-field digital mammography (FFDM) has largely replaced film-screen mammography in the US. Breast density assessed from film mammograms is strongly associated with breast cancer risk, but data are limited for processed FFDM images used for clinical care.

**Methods:**

We conducted a case-control study nested among non-Hispanic white female participants of the Research Program in Genes, Environment and Health of Kaiser Permanente Northern California who were aged 40 to 74 years and had screening mammograms acquired on Hologic FFDM machines. Cases (n = 297) were women with a first invasive breast cancer diagnosed after a screening FFDM. For each case, up to five controls (n = 1149) were selected, matched on age and year of FFDM and image batch number, and who were still under follow-up and without a history of breast cancer at the age of diagnosis of the matched case. Percent density (PD) and dense area (DA) were assessed by a radiological technologist using Cumulus. Conditional logistic regression was used to estimate odds ratios (ORs) for breast cancer associated with PD and DA, modeled continuously in standard deviation (SD) increments and categorically in quintiles, after adjusting for body mass index, parity, first-degree family history of breast cancer, breast area, and menopausal hormone use.

**Results:**

Median intra-reader reproducibility was high with a Pearson’s *r* of 0.956 (range 0.902 to 0.983) for replicate PD measurements across 23 image batches. The overall mean was 20.02 (SD, 14.61) for PD and 27.63 cm^2^ (18.22 cm^2^) for DA. The adjusted ORs for breast cancer associated with each SD increment were 1.70 (95 % confidence interval, 1.41–2.04) for PD, and 1.54 (1.34–1.77) for DA. The adjusted ORs for each quintile were: 1.00 (ref.), 1.49 (0.91–2.45), 2.57 (1.54–4.30), 3.22 (1.91–5.43), 4.88 (2.78–8.55) for PD, and 1.00 (ref.), 1.43 (0.85–2.40), 2.53 (1.53–4.19), 2.85 (1.73–4.69), 3.48 (2.14–5.65) for DA.

**Conclusions:**

PD and DA measured using Cumulus on processed FFDM images are positively associated with breast cancer risk, with similar magnitudes of association as previously reported for film-screen mammograms. Processed digital mammograms acquired for routine clinical care in a general practice setting are suitable for breast density and cancer research.

## Background

A large body of epidemiologic research indicates that mammographic density (the extent of the breast that appears radiopaque on a mammogram) is strongly associated with breast cancer risk [1–3]. Most of this evidence comes from studies that assessed breast density from film-screen mammograms acquired after screening mammography became widespread in the 1980s.

Over the last decade, conventional film mammography has largely been replaced with full-field digital mammography (FFDM). Both technologies use X-rays to produce an image of the breast; one image is captured directly on film, while the other is captured as digital data. In FFDM, the raw images are processed for viewing and interpretation by the breast imaging specialist. In addition to improving the image aesthetics and visualization of breast cancer, these processing algorithms, which differ by manufacturer, also reduce the size of the digital file [4]. The raw digital images for mammography are some of the largest diagnostic imaging files in clinical practice. Most mammography facilities store only the processed FFDM images for presentation and interpretation by the radiologist [5].

Only a few studies of the association of mammographic density with breast cancer risk have been conducted using FFDM images processed for clinical display [6, 7]. These studies suggest that the association between breast density assessed from FFDM images and breast cancer risk may be slightly weaker than associations generally observed for density assessed from film-screen mammograms. To our knowledge, only one study has reported results for processed images acquired using the Selenia Digital Mammography System machines manufactured by Hologic (Hologic, Inc., Marlborough, MA, USA) [7], which are the most commonly used FFDM machines in the US. Since the processing algorithms may change the appearance of dense tissue on the digital mammogram, the objective of this study was to determine whether percent density and dense area of the breast, assessed from processed digital images acquired from Hologic machines used in a general clinical setting are associated with breast cancer risk.

## Methods

### Setting

This study is ancillary to a genome-wide association study (GWAS) of mammographic density conducted among approximately 27,000 non-Hispanic white female participants of the Research Program in Genes, Environment and Health (RPGEH), who completed a health survey and provided a saliva sample for genotyping and who had a least one screening FFDM between 2003 and 2013. The RPGEH was developed and is administered by the Division of Research, Kaiser Permanente Northern California (KPNC). Briefly, the RPGEH resource enables research on the genetic and environmental determinants of common, age-related complex health conditions. The resource links together surveys, biospecimens, and derived data, with longitudinal data from electronic health records (EHRs) on a cohort of approximately 200,000 consenting adult KPNC members. Genome-wide genotyping has been performed on DNA extracted from saliva samples of more than 100,000 RPGEH participants enrolled before 2010 (RC2 AG036607).

### Mammograms

The EHR was used to identify screening mammograms on the study population. Processed FFDMs from 37 different mammography facilities, with one to five machines per facility, were obtained from the KPNC imaging archive. For women with a history of breast cancer, we obtained the closest pre-diagnostic FFDM after the RPGEH survey when available, or prior to the survey date otherwise, and selected the craniocaudal (CC) view of the unaffected breast (i.e., we used the left view for cases with cancer in the right breast and the right view for cases with cancer in the left breast). For control women, we randomly selected the right CC view for approximately 10 % to blind the reader to case-control status, and used the left CC view otherwise. We excluded women with bilateral breast cancer (n = 15), breast implants (n = 903), whose breasts were too large to be completely imaged on a single exposure (n = 245), or whose images were unreadable (n = 44) or unavailable (n = 625). FFDM images, in Digital Imaging and Communications in Medicine (DICOM) format, were de-identified and downsampled from a pixel size of 70 microns to a pixel size of 200 microns for transfer to the Stanford Radiology 3D and Quantitative Imaging Laboratory. Prior studies of scanned film mammograms have used larger pixel size [8], which would not be expected to influence computer-assisted density measurements on standard monitors that have lower resolution than the downsampled images.

### Density assessments

**Fig. 1:**
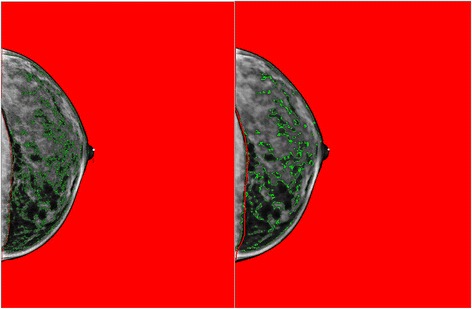
Representative full-field digital mammography image, with Cumulus dense tissue segmentation (*outlined in green*). On the *left* is the original image before downsampling and median filtering; on the *right* is the image after downsampling (from pixel size of 70 microns to a pixel size of 200 microns) and median filtering (with a radius of three pixels)

FFDMs acquired from Selenia Digital Mammography System machines manufactured by Hologic, Inc. for approximately 21,000 women were randomly assembled into 23 batches of up to 1100 images including 10 % random replicates for quality control. Density measurements were estimated with the Cumulus interactive threshold method [9]. We previously found that noise reduction of processed Hologic FFDM images to make them appear more film-like can significantly (*p* < 0.001) improve the reproducibility of readers with little prior experience applying Cumulus to processed FFDM images, and slightly increase the percent density measurements by about two percentage points. As readers gained experience over time, high levels of reproducibility (Pearson’s *r* >0.90) were attained on processed FFDM images with or without noise reduction. Here, we applied a median filter with a radius of three pixels [10] to all processed Hologic FFDM images (see Fig. 1 for a representative image both before and after downsampling and filtering). A single radiological technologist (RYL), trained in Cumulus assessments by MJY and JAL and blinded to case-control status, measured the total area of the breast and area of dense tissue using Cumulus6 (provided by MJY), which automatically detects the outer edge of the breast for most digital mammograms. The Cumulus software also calculated the percentage of the total breast area occupied by dense tissue (percent density).

### Cases and controls

This case-control study was nested among women between the ages of 40 and 74 years at Hologic FFDM. Breast cancer diagnoses were identified from the KPNC cancer registry, which reports to the California Cancer Registry and to the National Cancer Institute’s Surveillance, Epidemiology and End Results (SEER) program of cancer registries. The KPNC registry records information on all new primary cancers (except nonmelanoma skin cancer) diagnosed among KPNC members. Data elements and quality assurance measures are similar to SEER. Cases (n = 297) were women with a first primary, unilateral, invasive breast cancer diagnosed after a FFDM. Up to five controls (n = 1149) were selected at random from among women who matched the corresponding case on age at FFDM (exact year), calendar year at FFDM, breast laterality (left or right), and image batch number, and who were still under follow-up and without a history of breast cancer at the age of diagnosis in the matched case.

### Covariates

Age at mammogram was determined based on date of birth (demographic database) and date of mammogram (mammography database). We used the body mass index (BMI) measured at the patient visit closest to mammogram date when available from the EHR, and computed from self-reported height and weight on the RPGEH survey otherwise. The RPGEH survey provided information on parity and history of breast cancer in a first-degree family member. The KPNC pharmacy database, which records all dispensed outpatient and inpatient prescriptions, was used to determine use of menopausal hormones within the 2 years prior to FFDM.

### Statistical methods

Conditional logistic regression was used to estimate odds ratios (ORs) for breast cancer associated with percent density (PD) and with dense area (DA). PD and DA were categorized into quintiles based on their distributions in control women. We applied a square-root transformation to PD and cube-root transformation to DA to obtain normal distributions, and we modeled PD and DA as continuous variables in units of the standard deviation (SD) in controls. To maximize adjustment for BMI (kg/m^2^), it was modeled as both a categorical variable (<25, 25–29, 30–34, 35–39, 40+) and as a continuous variable. Total breast area (cm^2^) was modeled as a continuous variable to facilitate comparison to previous studies [6]. Parity was categorized as nulliparous, parous, or missing. History of breast cancer in a first-degree family member was categorized as yes or no. The use of menopausal hormones was categorized as none, estrogen alone, or estrogen plus progestin. To examine whether associations between measures of density and breast cancer risk varied by menopausal status, we conducted sub-analyses restricted to women aged 50+ years as a surrogate for postmenopausal status. The number of women younger than 50 years of age was too small for meaningful results.

The study was approved by Institutional Review Boards at Kaiser Permanente and Stanford University. All study participants provided written informed consent.

## Results

This study included 297 invasive breast cancer cases and 1149 matched controls (Table 1). Fewer than 5 % of cases and controls were younger than 50 years of age at reference date. Compared to controls, a smaller proportion of cases had a BMI less than 25 and a slightly larger proportion had a family history of breast cancer or were nulliparous. Density measurements using Cumulus on processed Hologic FFDM images were highly reproducible. The median intra-reader reproducibility, estimated by Pearson’s *r*, was 0.956 (range 0.902 to 0.983) across 23 image batches read over a period of 7 months. The overall mean was 20.02 (SD, 14.61) for PD and 27.63 cm^2^ (SD, 18.22 cm^2^) for DA.

**Table 1:** Characteristics of 297 cases and 1149 controls

	Cases		Controls	
	N = 297	%	N = 1149	%
Age at mammogram				
Mean (SD)	63.79	7.21	63.95	7.00
Categories:				
40–49 years	9	3.0 %	33	2.9 %
50–59 years	67	22.6 %	238	20.7 %
60–69 years	149	50.2 %	600	52.2 %
70+ years	72	24.2 %	278	24.2 %
Body mass index (kg/m^2^)				
Mean (SD)	28.71	6.88	27.48	5.88
Categories:				
24 or less	97	32.7 %	471	41.0 %
25–29	99	33.3 %	347	30.2 %
30–34	53	17.8 %	213	18.5 %
35–39	22	7.4 %	71	6.2 %
40+	26	8.8 %	47	4.1 %
First-degree family history of breast cancer				
No	257	86.5 %	1058	92.1 %
Yes	40	13.5 %	91	7.9 %
Use of postmenopausal hormones^a^				
None	237	79.8 %	939	81.7 %
Estrogen alone	28	9.4 %	106	9.2 %
Estrogen + progestin	32	10.8 %	104	9.1 %
Parity				
Nulliparous	18	6.1 %	65	5.7 %
Parous	229	77.1 %	916	79.7 %
Missing	50	16.8 %	168	14.6 %
Percent density^b^				
Mean (SD)	22.00	13.81	19.51	14.77
Quintiles:				
Q1: 6.08 or less	40	13.5 %	229	19.9 %
Q2: 6.09–11.94	49	16.5 %	230	20.0 %
Q3: 11.95–20.04	60	20.2 %	230	20.0 %
Q4: 20.05–31.56	69	23.2 %	230	20.0 %
Q5: 31.57+	79	26.6 %	230	20.0 %
Area of density^b^				
Mean (SD)	32.66	20.13	26.33	17.47
Quintiles:				
Q1: 11.95 or less	30	10.1 %	229	19.9 %
Q2: 11.96–19.09	45	15.2 %	230	20.0 %
Q3: 19.10–26.96	66	22.2 %	230	20.0 %
Q4: 26.97–37.78	68	22.9 %	230	20.0 %
Q5: 37.79+	88	29.6 %	230	20.0 %
Total breast area				
Mean (SD)	175.92	81.23	167.67	74.01

^a^Within 2 years prior to mammogram date

^b^Measures are untransformed. Quintiles calculated from distribution of controls

Odds ratios (ORs) for the association between PD and breast cancer risk are shown in Table 2. The results were similar in models adjusted for BMI (model 1); BMI, parity, first-degree family history, and menopausal hormone use (model 2); or BMI, parity, first-degree family history, menopausal hormone use, and breast area (model 3) indicating little confounding by parity, first-degree family history, hormone use, and breast area in our data. Among all women, those in the highest quintile of PD had a significantly increased risk of breast cancer (OR, 4.88; 95 % confidence interval (CI), 2.78–8.55) compared to women in the lowest quintile, after adjusting for BMI, parity, first-degree family history, hormone use, breast area, and matching factors. The OR for each SD increment was 1.70 (95 % CI, 1.41–2.04). The association appeared to be similar in analyses restricted to women aged 50+ years.

**Table 2:** Percent density, overall and for women age 50+ at mammogram

Quintile group	Frequency		Model 1^a^		Model 2^b^		Model 3^c^	
	Case	Control	OR	95 % CI	OR	95 % CI	OR	95 % CI
Percent density, overall (297 cases and 1149 controls)								
Q1: 6.08 or less	40	229	1.00	Ref	1.00	Ref	1.00	Ref
Q2: 6.09–11.94	49	230	1.41	0.87–2.29	1.41	0.86–2.29	1.49	0.91–2.45
Q3: 11.95–20.04	60	230	2.37	1.44–3.89	2.38	1.44–3.91	2.57	1.54–4.30
Q4: 20.05–31.56	69	230	3.08	1.87–5.06	2.95	1.79–4.88	3.22	1.91–5.43
Q5: 31.57+	79	230	4.45	2.62–7.56	4.36	2.55–7.43	4.88	2.78–8.55
Percent density^d^			1.66	1.39–1.97	1.64	1.38–1.96	1.70	1.41–2.04
Percent density, women age 50+ (288 cases and 1116 controls)								
Q1: 6.07 or less	40	223	1.00	Ref	1.00	Ref	1.00	Ref
Q2: 6.08–11.71	48	223	1.39	0.85–2.26	1.39	0.85–2.27	1.47	0.89–2.43
Q3: 11.72–19.71	57	223	2.23	1.35–3.68	2.24	1.36–3.71	2.43	1.45–4.08
Q4: 19.72-30.97	66	223	2.97	1.79–4.92	2.87	1.72–4.76	3.13	1.85–5.31
Q5: 30.98+	77	224	4.22	2.49–7.16	4.16	2.45–7.08	4.65	2.66–8.14
Percent density^d^			1.64	1.38–1.95	1.63	1.36–1.94	1.68	1.40–2.02

^a^Adjusted for matching factors (age at FFDM, laterality, density batch number), BMI (continuous), and BMI (categorical)

^b^Adjusted for matching factors, BMI (categorical and continuous), parity, first-degree family history, and hormone use

^c^Adjusted for matching factors, BMI (categorical and continuous), parity, first-degree family history, hormone use, and breast area (continuous)

^d^ORs for square root of percent density (continuous) per standard deviation unit

Odds ratios (ORs) for the association between DA and breast cancer risk are shown in Table 3. Among all women, those in the highest quintile of DA had a significantly increased risk of breast cancer (OR, 3.48; 95 % CI, 2.14–5.65) compared to women in the lowest quintile, after adjusting for BMI, parity, first-degree family history, hormone use, breast area, and matching factors. The OR for each SD increment was 1.54 (95 % CI, 1.34–1.77). The association was slightly stronger in analyses restricted to women aged 50+ years.

**Table 3:** Area of density, overall and for women age 50+ at mammogram

Quintile group	Frequency		Model 1^a^		Model 2^b^		Model 3^c^	
	Case	Control	OR	95 % CI	OR	95 % CI	OR	95 % CI
Area of density, overall (297 cases and 1149 controls)								
Q1: 11.95 or less	30	229	1.00	Ref	1.00	Ref	1.00	Ref
Q2: 11.96–19.09	45	230	1.44	0.86–2.42	1.45	0.86–2.43	1.43	0.85–2.40
Q3: 19.10–26.96	66	230	2.63	1.59–4.34	2.56	1.55–4.22	2.53	1.53–4.19
Q4: 26.97–37.78	68	230	2.86	1.74–4.69	2.88	1.75–4.75	2.85	1.73–4.69
Q5: 37.79+	88	230	3.53	2.18–5.71	3.45	2.12–5.60	3.48	2.14–5.65
Dense area^d^			1.52	1.33–1.74	1.51	1.32–1.73	1.54	1.34–1.77
Area of density, women 50+ (288 cases and 1116 controls)								
Q1: 11.80 or less	29	223	1.00	Ref	1.00	Ref	1.00	Ref
Q2: 11.81-18.71	44	223	1.48	0.88–2.51	1.49	0.88–2.52	1.47	0.87–2.50
Q3: 18.72-26.66	65	223	2.73	1.64–4.54	2.68	1.61–4.46	2.66	1.59–4.43
Q4: 26.67-37.40	66	223	2.96	1.79–4.91	2.96	1.79–4.92	2.93	1.76–4.87
Q5: 37.41+	84	224	3.54	2.17–5.79	3.49	2.13–5.71	3.51	2.15–5.76
Dense area^d^			1.52	1.32–1.74	1.51	1.32–1.73	1.53	1.33–1.76

^a^Adjusted for matching factors (age at FFDM, laterality, density batch number), BMI (continuous), and BMI (categorical)

^b^Adjusted for matching factors, BMI (categorical and continuous), parity, first-degree family history, and hormone use

^c^Adjusted for matching factors, BMI (categorical and continuous), parity, first-degree family history, hormone use, and breast area (continuous)

^d^ORs for cube root of dense area (continuous) per standard deviation unit

## Discussion

Studies of mammographic density as a risk factor or potential surrogate of breast cancer risk have historically used film-screen mammograms. Now that FFDM has replaced film-screen mammography as the most common breast imaging modality, it is critical to determine whether density measured from digital images, especially processed digital images routinely archived for clinical care, are suitable for research purposes. Our study has shown that breast density measured on processed digital mammograms acquired from multiple Hologic units in general practice settings is reproducible and strongly associated with risk of invasive breast cancer. The associations with breast cancer risk were slightly stronger for PD than for DA, as has been found in studies using film-screen mammograms [3]. Our findings indicate that processed Hologic FFDM images, which are widely used for clinical care in the US, are suitable for determining breast density for risk assessment and breast cancer research.

The FDA approved the first clinical digital mammography system for use in the US in early 2000 [4]. The diagnostic accuracy is similar for film-screen and digital mammography for all women combined. However, digital mammography has been found to be better at detecting breast cancers in women who are pre- or perimenopausal, under age 50 years, and have dense breasts [11]. It is unknown why digital mammography performs better for women with dense breasts, but one possible explanation may be that proprietary imaging algorithms enhance contrast between dense tissue and adjacent structures [12]. Radiologists have observed that breasts appear to be less dense on processed digital images than on film mammograms [13]. Alterations of the appearance of dense tissue on digital mammograms could explain differences in the association with breast cancer risk compared to film mammograms or different FFDM manufacturers.

To our knowledge, only one other study has examined breast density measured using Hologic FFDM images in relation to breast cancer risk. Fowler et al. [7] used both Cumulus, as well as an automated method, to measure PD from raw and processed images from 192 women with breast cancer and 358 matched controls. The mean PD was similar but slightly higher for processed than raw images; mean PD for processed vs. raw was 18.1 vs. 15.0 for cases and 16.9 vs. 13.6 for controls. The reported associations for Cumulus measures of PD were similar for processed and raw FFDM images, but the magnitude of the associations were weaker than in our study. The adjusted OR reported for quartile 4 vs. 1 was 1.95 for processed and 2.04 for raw images. The adjusted OR for a one SD increment of PD was 1.22 for processed and 1.21 for raw images.

In an early study of FFDM images from General Electric (GE) Senographe mammography machines (General Electric Healthcare, Chicago, IL, USA), Nagata et al. [14] used an automated density assessment method to compare PD for 75 breast cancer cases and 289 controls from Gifu City, Japan. Among postmenopausal women, the mean PD was 18.2 for cases and 16.2 for controls. The adjusted OR was 4.2 when comparing 50–100 % dense to 0 % dense. More recently, Vachon et al. [6] compared PD assessments from raw and processed FFDM images from a single GE Senographe mammography machine. The study included 180 matched case-control pairs and density was assessed by a single reader using the Cumulus method. They found that intra-reader reproducibility was high for both raw and processed images (*r* = 0.92 and 0.87, respectively). Readings from raw and processed images were strongly correlated (*r* = 0.82), and they had similar means and standard deviations. For raw and processed images, respectively, the mean PD was 21.3 and 22.5 for cases, and 17.7 and 19.8 for controls. PD measured from raw and processed GE digital images also showed similar associations with breast cancer risk (the adjusted OR for quartile 4 vs. 1 was 3.99 for processed and 5.17 for raw images). In another recent study of FFDM images from GE machines [15], Eng et al compared six density assessment methods, including Cumulus. Prior to the Cumulus assessments, the raw FFDM images (414 breast cancer cases and 685 controls) were converted into film-like images. The intra-reader reproducibility for PD was 0.90. Among controls, the median PD for Cumulus was 6.8. The adjusted OR comparing quintile 5 vs. 1 was 3.38, and the adjusted OR for each SD increment of PD was 1.58. Thus, the associations with breast cancer risk, intra-reader reproducibility, and PD distributions found in our study of processed FFDM images were similar to previous studies using GE images.

These initial results from density studies using FFDM images are consistent with results from earlier studies using film-screen mammograms. In a recent meta-analysis of 13 case-control studies of mammographic density and breast cancer risk using density measures from film-screen mammograms, the summary OR for each SD increment of PD was 1.52 for premenopausal women and 1.53 for postmenopausal women [3]. The meta-analysis finding of stronger associations with PD than with DA is also similar to the pattern observed in our study of processed Hologic mammograms.

Our study has several strengths. We included FFDMs from multiple different mammography facilities acquired over a 10-year period during 2003–2013, and thus our results are likely to be generalizable to other contemporary multi-institutional studies of Hologic FFDM images. A single radiological technologist conducted all assessments using the operator-assisted Cumulus method, which is the most widely used density measurement method for research studies. We examined both percent density and dense area and were able to adjust for age, BMI, parity, first-degree family history of breast cancer, and use of hormonal therapy – all factors associated with both breast density and breast cancer risk.

The study also had some limitations. All study participants are members of the Kaiser Permanente Northern California health plan. While the membership is demographically quite similar to that of the general population in northern California, it does slightly under-represent individuals at the extremes of the socioeconomic spectrum. Nonetheless, members get virtually all their healthcare from the plan, so all mammograms of interest were available to the study.

## Conclusions

Our study is one of the first to demonstrate that density assessments using Cumulus on processed Hologic FFDM images for clinical display can be highly reproducible and strongly associated with breast cancer risk. These findings add to the growing evidence that FFDM images routinely acquired and stored for clinical care in general practice settings are an appropriate resource that can be leveraged for large-scale research studies of breast density and cancer risk. Our results suggest that the magnitude of associations using FFDM images acquired from Hologic machines, the most common type in the US, are similar to those from GE machines and to film-screen mammography [1, 2]. Further studies are needed to confirm these findings. Given that FFDM hardware and software vary by manufacturer and evolve over time, additional studies using other FFDM systems, such as those manufactured by Fuji Medical Imaging and Fischer Medical Systems, are also needed. Further studies are also needed to validate the emerging, fully automated methods to measure density.

## Abbreviations

BMI: body mass index
CC: craniocaudal
CI: confidence interval
DA: dense area
DICOM: Digital Imaging and Communications in Medicine
EHR: electronic health records
FFDM: full-field digital mammography
GE: General Electric
GWAS: genome-wide association study
KPNC: Kaiser Permanente Northern California
OR: odds ratio
PD: percent density
RPGEH: Research Program in Genes, Environment and Health
SD: standard deviation
SEER: Surveillance, Epidemiology and End Results
